# The Climate of the United States and Its Endemic Influences

**Published:** 1842-03-26

**Authors:** 


### BIBLIOGRAPHICAL NOTICES.

*The Climate of the United States and its Endemic Influences. Based chiefly on the Records of the Medical Department and Adjutant General's Office, United States Army.* By SAMUEL FORRY, M. D. New York. 1842. 8vo. pp. 378.

A very happy order of the department of Washington enjoined upon the surgeons of the different military posts scattered throughout the United States, to collect careful observations of the most prominent meteorological phenomena observed at their different stations, and to transmit them to Washington with their reports of sickness and mortality. These observations are not of course as complete as those which could be instituted by scientific men exclusively occupied with meteorology, instead of medical officers who have other and more pressing duties to perform. They are complete enough, however, to furnish many valuable data. These have been carefully collected by Dr. Forry, who was for some time attached to the medical bureau of the army, at Washington, under the charge of the able surgeon-general Dr. Lawson. This volume contains the results of his laborious and most ably executed enterprise.

As a volume of medical statistics, it is the first published in the United States, and has such internal evidence of authenticity that it must remain as the basis of future observations, which will by its aid become more definite and much more easy. The work is well and carefully drawn up, and the deductions of the author are quite within the limits of his facts, perhaps they might have been carried still further without diminishing their value as accurate statistical data. As a vast collection of documents, the book will be much read, and more consulted by those who take an interest in the important subject of the influence of the climate of the United States on human life. A little more simplicity of style and expression would have been better adapted to a work of this severely philosophical character, but we are not disposed to cavil at a trifling disadvantage, where the tenor of the whole is decidedly good—almost unobjectionable.

The work commences with an essay on Climatology, especially in connexion with the climate of the United States, which, with the exception of the southern portion, belongs to those countries in which the climate is excessive, or characterized by extreme differences of temperature. This disappears, as just mentioned, at the south eastern extremity, and is much less perceptible along the Pacific than the Atlantic coast. It has, on the whole, a very close similarity to the climate of China; that is, at the corresponding coast of Asia. As examples of extremes we have Fort Snelling, where the same temperature of the winter is 15° 95′, and of the summer 72° 75′; at Key West that of the winter is 70° 05′, of the summer 81° 39′.

The question of the stability of climates in modern and ancient times is also touched upon, but not solved. The opinion of Arago that the climate of the world has been gradually getting milder for the last ten or twenty centuries is probably correct.

The next division of his subject leads the author to the strictly medical portion of his work, the endemic influences of the United States. The topography and peculiar circumstances relative to the posts on the northern chain of lakes are first described. Those near the most northern limits, at Mackinac, for example, are extremely salubrious, while intermittents and remittents are met with at the posts on the southern borders of some of the lakes. At the most healthy, the mortality is only 6-10 per cent.; but 1 4-10 and 1 5-10 where autumnal fevers prevail.

The second class includes the posts on the New England coast. In Boston Harbor the deaths are 1 3-10 per cent. from medical causes (excluding casualties.) Pleuritis and pneumonia now prevail instead of the catarrhal affections of the lakes. The post at New Port is pre-eminently healthy; no epidemics have prevailed there.

The third class includes the northern posts, remote from both the sea and lakes. These were very healthy; at West Point only 2 8-10 per 1000; at two other stations, where there are only troops of the line, 5-10 per cent. On the Mississippi and Missouri the mortality is higher, miasmatic causes here operating; at Cantonment Leavenworth 1 2 10 from medical diseases.

The middle division of the United States, according to Dr. Forry's classification, begins with Pennsylvania. Most of the posts are here in the alluvial parts, on the banks of the rivers where malaria prevails. The mortality was at one post (Fort Munroe) near Norfolk, as high as 3 2-10, exclusive of casualties and epidemic cholera. Near Savannah the mortality is 5 5-10; easily explained from the low rice fields near the town, and the prevalence of autumnal fevers.

The second class of this division embraces the interior posts. Near St. Louis, exclusive of cholera, the deaths are 3 5-10 per cent.; the barracks are on the banks of the Mississippi river, and many autumnal fevers prevail. At Fort Gibson (Arkansas) the mortality is 4 5-10, excluding cholera and casualties. Intermittents are more prevalent here than at any other post; sometimes, as in 1834, the autumnal fevers are very malignant. At other posts the mortality varies according to situation from two per cent. to 4 7-10.

The general results of this class of posts (exclusive of cholera and accidents) give a mortality of 3 6-10. Almost all of them are in malarious districts.

The third division includes the low countries on the southern border of the United States. Near Augusta, in a favourable situation on the sand hills, the mortality is 3 5-10. At Fort Mitchell (Alabama) nearly three per cent. At Baton Rouge (Louisiana) the mortality was enormous, 7 2-10, partly from endemic causes, and partly from most laborious duties required of the soldier. At New Orleans the mortality is less, 4 2-10. At both these points the chief cause of death is malignant remittent, otherwise called congestive fever, or, by the inhabitants, sometimes cold plague. On Lake Ponchatrain we have a healthy post, like the rest of its shores, with a mortality of about two per cent., but another post nearer the marshes of the Mississippi give a mortality of more than five per cent. Below New Orleans on the river it is less; mortality at the two posts at Plaquemine bend give 4 4-10 and 5 5-10 from medical causes.

The second class of this division includes the forts and posts in East Florida. The mortality varies from 1 8-10 to 9 8.10, (Key West,) chiefly from medical causes.

The classification ends with the temporary posts occupied during the Florida war, but of course of little or no value for a statistician.

It is very obvious, therefore, that the soldier rarely dies at his post of a medical disease, except from malarious causes, in the southern parts of the U. States. Is his health, therefore, equal to that of the civilian? There is, however, a source of fallacy in these documents: a soldier enlists for three years, and besides often deserts before the expiration of his service, and is sometimes discharged for ill health, and other causes. We have met with many, both officers and soldiers, who in reality fell victims to the climate of unhealthy posts, but died long after leaving them. This is especially the case with those from Florida, where the exposure is extremely great. The average age of soldiers is also favourable, so far as their power of resistance to disease is concerned. We may infer, therefore, that unhealthy climates are much more injurious to the life of the soldier than they would appear to be, because death follows much more frequently the slow rather than the rapid action of malarial poison (unless highly concentrated,) dropsy, phthisis, chronic dysentery, &c. being the frequent slow terminations. In the more healthy stations, however, the mortality may be considered the real average for persons placed under circumstances of the military as regards age, &c. On the whole, however, it is perfectly shown by the researches of Villermé that the military life is destructive even in peace, and the mortality amongst them is a little higher than civilians under the same circumstances.

The next section of the work is the general deductions; we extract the most interesting remarks. The average of catarrh, pleuritis, and pneumonia is much lower in the northern than the middle posts. So also as regards phthisis, the same result holds good, which is not exactly in accordance with ordinary belief. Dr. Forry accounts for it, in part at least, by the predisposition produced by the high temperature of summer, followed by the cold of winter. The author next states the advantages of Key Biscayno on the eastern coast of Florida, as a residence for consumptive patients; probably as good as other points for permanent stay during the winter, but movement and travelling in incipient phthisis are indispensable. Rheumatism, it would seem, is more frequent in the northern than in the middle, and still more than in the southern states. The predominant diseases of the southern section are, of course, the different forms of remittent and intermittent fevers, with various bowel affections.

The general deductions are not limited to those which are immediately dependant upon the results of the statistics of the United States service. The excellent statistics of the British army are analyzed, and compared with the documents published in the beginning of the volume. The field is therefore a wide one, and if the author has been less happy in his analysis than we might have desired, it was probably owing to the want of time and research necessary for turning so large a mass of documents to profit. The study of the French writers on statistics, especially Villermé and Parent du Chatelet, would have been a good preparation for the task, that is, their later memoirs, for their earlier essays are marked with the same faults as that to which we are alluding. Our surprise is not, however, that the work should not be faultless, but that so laborious and apparently ungrateful a task should have led to such important results, furnishing a starting point for future writers which was previously not attainable.

